# Macrophage-Driven Inflammation in Metabolic Osteoarthritis: Implications for Biomarker and Therapy Development

**DOI:** 10.3390/ijms24076112

**Published:** 2023-03-24

**Authors:** Kelly Warmink, Prateeksha Vinod, Nicoline M. Korthagen, Harrie Weinans, Jaqueline L. Rios

**Affiliations:** 1 Department of Orthopedics, University Medical Center Utrecht, 3584 CX Utrecht, The Netherlands; 2 Department of Equine Sciences, Faculty of Veterinary Medicine, Utrecht University, 3584 CL Utrecht, The Netherlands; 3 Department of Biomechanical Engineering, TU Delft, 2628 CD Delft, The Netherlands

**Keywords:** osteoarthritis, obesity, inflammation, macrophages, metabolic syndrome

## Abstract

Osteoarthritis (OA) is a common and debilitating joint disorder that leads to progressive joint breakdown and loss of articular cartilage. Accompanied by a state of low-grade inflammation, its etiology extends beyond that of a wear-and-tear disease, and the immune system might have a role in its initiation and progression. Obesity, which is directly associated with an increased incidence of OA, alters adipokine release, increases pro-inflammatory macrophage activity, and affects joint immune regulation. Studying inflammatory macrophage expression and strategies to inhibit inflammatory macrophage phenotype polarization might provide insights into disease pathogenesis and therapeutic applications. In pre-clinical studies, the detection of OA in its initial stages was shown to be possible using imaging techniques such as SPECT-CT, and advances are made to detect OA through blood-based biomarker analysis. In this review, obesity-induced osteoarthritis and its mechanisms in inducing joint degeneration are summarized, along with an analysis of the current developments in patient imaging and biomarker use for diagnostic and therapeutic strategies.

## 1. Introduction

Osteoarthritis (OA) is the most prevalent joint disease, affecting around 250 million people worldwide [1]. The main risk factors associated with the development of OA include aging, obesity, sex (higher prevalence in women), joint injury, genetics, and bone deformities [2]. Between 1990 and 2013, OA was the third most rapidly rising condition after diabetes and dementia [3], and the number of patients with OA is predicted to double by 2040 [3,4]. During the development and progression of OA, the whole joint can be affected, including the synovium, Hoffa’s fat pad, subchondral bone, articular cartilage, ligaments, joint capsule, and the periarticular muscles, leading to symptoms such as pain, joint stiffness and loss of mobility, hence severely impacting quality of life [5,6,7].

In response to damage in the joint, local immune processes come to play, resulting in chronic, slow, and low-grade inflammation leading to joint degeneration. This low-grade inflammation differentiates OA from other joint diseases like rheumatoid arthritis (RA), as individuals with OA display much lower levels of inflammatory factors in synovial fluid and blood compared to patients with RA [8,9,10]. In cartilage, tissue hallmarks of inflammation include excessive cytokine expression and degrading enzymes, like matrix metalloproteinases (MMPs) and a disintegrin and metalloproteinase with thrombospondin motifs (ADAMTs) [11]. Moreover, in the joint synovium, inflammation is marked by an imbalance between pro-inflammatory M1-like macrophages and anti-inflammatory M2-like macrophages during the early stages of joint inflammation and injury [12]. In the healthy joint, macrophages in the synovium predominantly show an M2-like phenotype, while during OA development, a shift towards M1-like macrophages is observed, suggesting a correlation between macrophage polarization and OA [13,14].

M1 and M2-like macrophages are thought to be two phenotype extremes in the spectrum of in vivo polarization states, making elucidation of molecular mechanisms contributing to polarization pathways complex. Nevertheless, several mechanisms in OA have been identified as contributing to M1-like pro-inflammatory macrophage polarization; for example, lipopolysaccharide (LPS) is found to polarize macrophages via activation of nuclear factor (NF)κB and mitogen-activated protein kinase (MAPK), while interferon (IFN)-γ and tumor necrosis factor (TNF) activate the Janus kinase/signal transducer and activator of transcription (JAK/STAT) pathway in pro-inflammatory polarization [15,16]. Other signaling mechanisms that are involved in macrophage pro-inflammatory reprogramming include c-Jun N-terminal kinase (JNK), phosphatidylinositol 3-kinase (PI3K)/Akt, Notch, and hypoxia-dependent intracellular pathways [17]. M2-like macrophages in the synovium and infiltrating monocytes have been shown to subsequently differentiate to M1-like macrophages and contribute to synovial inflammation and thickening of the synovium [8,14]. This imbalance between M1-like and M2-like phenotypes is thought to contribute to OA development by triggering a prolonged state of local inflammation, with pro-inflammatory cytokines inducing synovitis, loss of cartilage function, osteophyte formation, and ultimately, joint degradation [8,18,19].

Based on the main disease driver, six OA phenotypes have been proposed: cartilage-driven, synovitis inflammatory phenotype, subchondral bone phenotype, traumatic injury-driven, aging-driven, and finally, the metabolic phenotype [20,21]. Three of the proposed phenotypes are determined by the joint tissue that dominates the disease, e.g., cartilage degeneration, synovial inflammation, and subchondral bone changes (either bone volume loss or sclerosis). Traumatic injury is proposed as a phenotype as trauma to the joint is associated with OA development [20]. Furthermore, the aging phenotype is caused by an imbalance in cartilage maintenance and tissue development via chronic accumulation of senescent cells in joint tissues [22]. Lastly, the metabolic OA (MetS-OA) phenotype is driven by metabolic syndrome (MetS) [23,24]. The metabolic syndrome results from a combination of obesity, diabetes, insulin resistance, hypertension, and dyslipidemia, and all these factors have individually been linked to OA [25]. Firstly, obesity is known to be a strong risk factor for OA, which is not fully explained by the increase in weight and mechanical stress, as non-weight-bearing hand joints have a similar increased risk for OA development with obesity [26,27]. In addition, OA is associated with hypertension and type 2 diabetes, independent of age and BMI [28,29]. Both lipid and glucose dysregulation are shown to have a negative effect on cartilage health and are associated with knee OA independent of obesity [30,31,32].

The occurrence of the metabolic OA phenotype is thought to increase due to the high incidence of obesity in populations arising from a sedentary lifestyle and high-fat and high-sugar diets, amongst other causes [33,34]. In addition, the low-grade systemic inflammation associated with obesity directly affects macrophages and other components of the immune system. This has led to increased interest in identifying biomarkers exhibited by these macrophage phenotypes to understand disease pathogenesis and to find potential treatment opportunities. This review summarizes the role of obesity-driven macrophage activation in OA development and progression and its implications for improved diagnosis and treatment opportunities.

## 2. The Role of Macrophages in Osteoarthritis

Activation of the innate immune system occurs via pattern-recognition receptors (PRRs), which can recognize damage-associated molecular patterns (DAMPs) [8]. These DAMPs, released during tissue damage and injury, such as in OA development, trigger a cascade of inflammatory responses via the recruitment of inflammatory mediators and immune cells. DAMPs interact with Toll-like receptors (TLRs), a class of PRRs, leading to heightened expression of pro-inflammatory cytokines via transcription factors such as NF-κB and activator protein 1 (AP-1), which upregulate inflammatory cytokines like interleukin (IL)-1β and TNF, and degrading enzymes [35,36]. For example, TLR 2 and 4 are shown to be upregulated in OA cartilage lesions compared to normal cartilage and compared to cartilage far away from the lesion area [37]. Thus, constant or disturbed activation of these mechanisms can cause prolonged chronic inflammation, as seen in OA [38]. Similarly, the complement system, upon activation, induces a complement cascade with its effector proteins complement component (C)3a and C5a promoting inflammation via the activity of pro-inflammatory leukocytes and the membrane attack complex (MAC) [8,39]. Complement activation in OA is induced via the release of factors from the damaged cartilage, such as components of the extracellular matrix like fibromodulin, collagen type II, fibronectin as well as DAMPs, calcium crystals, apoptotic cells, and their cell debris, leading to sustained inflammation [40,41,42]. Compared to healthy synovium, the OA synovium was found to express higher levels of complement effectors, increased production of matrix-degrading enzymes like matrix metalloproteinases (MMP)s, and inflammatory mediators, like macrophage colony-stimulating factor (M-CSF) and cyclooxygenases [41,43].

DAMPs and the complement system can activate innate immune cells in the synovium, such as neutrophils, synovial mast cells, natural killer cells, and monocytes [38]. Neutrophils, one of the first responders to joint injury, are a player in OA progression through mechanisms such as induced tissue degeneration via secretion of degradative protease neutrophil elastase, osteophyte development, and enhanced release of inflammatory cytokines and chemokines [44]. Neutrophils also interact with other innate cells, such as Natural killers (NK), to promote OA. NK cells, which are cytotoxic lymphocytes, are found in inflamed synovial fluid due to neutrophil activity and are thought to lead to joint damage and OA establishment [45,46]. Other early responses to joint injury/damage implicated in OA are synovial mast cells which are also thought to play an important role in synovial inflammation. This could be via its attraction of other immune cells in response to cytokine or chemokine release, direct cell-to-cell contact, and other mechanisms like activation of fibroblasts, and stimulation of angiogenesis, ultimately causing inflammation and joint degradation [47].

Monocytes, key regulators of tissue repair, fibrosis, and regeneration, differentiate into macrophages, releasing pro-inflammatory factors at the lesion site [48]. Macrophages are known to have various polarization states, each identified by different activation markers [49]. In vitro, M1-like macrophages produce high levels of pro-inflammatory cytokines, such as IL-1β, IL-12, and TNF [14,50]. Alternatively, M2-like polarization occurs in response to cytokines such as IL-4, IL-13, IL-10, IL-33, and transforming growth factor (TGF)-β, and to induce polarization in vitro, IL-4 and IL-13 are used most often [14,51]. M2-like cytokine production can vary but often includes IL-10 and TGF-β [14,51].

Within a healthy synovial joint, macrophages reside in the synovium. They release factors such as proteases, cytokines, and reactive oxygen species (ROS), mediating inflammation, repair, and regeneration in the joint [52,53]. In the absence of threat, resident tissue macrophages, such as in the synovium, exist predominantly in their M2-like phenotype [54]. However, following inflammation or tissue damage, the M2-like macrophages switch their phenotype into M1-like macrophages [14,15]. This phenotype transformation affects tissue homeostasis, leading to disease, such as OA, its onset, progression, as well as chronic inflammation. Transcription factor-mediated polarization and signaling by signal transducer and activator of transcription (STATs), which drives M1 polarization in response to cytokines, NF-κB, peroxisome proliferator-activated receptor (PPAR)-γ in adipose tissue, are also crucial regulators of the M1 and M2 phenotype switch [16,55].

In studies about obesity, fatty acids, and TNF have been shown to induce M1 polarization along with obesity-induced hypoxia [56,57,58]. Adipose tissue from obese subjects shows a lower oxygen consumption, and this hypoxic state may increase the expression of cytokines such as IL-6 and IL-1β in adipocyte resident macrophages along with enhanced saturated fatty acid-induced pro-inflammatory cytokine production leading to an inflammatory shift of the adipose tissue-resident macrophage phenotype [56,57,58].

In addition, it is hypothesized that during the progression of OA, macrophage phenotype in the joint itself is switched, initiating a feedforward loop with continual release of pro-inflammatory factors, triggering chronic inflammation in attempts to repair the damaged tissue [52,53]. Bailey et al. (2020) noted a shift in macrophage M1/M2 ratio following joint damage in mice, leading to the dominance of M1-like macrophages and a reduction of M2-like macrophages in the synovium [59]. In particular, pro-inflammatory macrophage polarization is associated with triggering other tissue responses seen in OA, such as synovial inflammation. Synovitis is observed in the early stages of OA [60,61], and it has been reported to precede the development of radiographic knee osteoarthritis [62,63]. For instance, patients with early OA have greater macrophage cell infiltration, blood vessel formation, and a significantly higher number of cells producing inflammatory cytokines such as TNF and IL-1β when compared to patients with late OA [64]. Nevertheless, on magnetic resonance imaging (MRI), synovitis is also observed in the later stages of OA [60]. Synovitis seems to contribute towards OA progression, with a higher synovitis score corresponding to an increased risk of OA incidence, an increase in chronic pain, and joint inflammation [15,65]. When investigating the ratio of M1-like and M2-like macrophages in OA knees and healthy control knees, synovial fluid macrophages and peripheral blood monocytes showed higher M1-like versus M2-like markers in patients with OA compared to healthy controls [13]. Flow cytometry and RT-qPCR showed a significantly higher expression of the CD11c marker (M1) in the synovial fluid of knees with OA compared to CD206 expression (M2), and the ratio of M1 and M2 macrophages was positively correlated with the level of the Kellgren-Lawrence grade in knee OA, implying high OA severity [13].

Macrophage activity in OA has led to studies investigating whether cartilage and joint health benefit from macrophage modulation [66]. Studies on therapeutic applications have focused on developing alternative designs to inhibit pro-inflammatory signal transduction carried out by the innate immune system [14,67]. Building upon previously established knowledge of TGF-β in inducing osteophyte formation in murine joints, macrophages were identified to be crucial intermediaries in TGF-β induced osteophytes and chondrogenesis [19,68]. Depletion of synovium macrophages in normal murine knee joints via injection of clodronate-laden liposomes resulted in significantly decreased osteophyte formation (by 70%) despite TGF-β stimulation [19]. In addition, supplementation of corticosteroids, such as dexamethasone, to synovial explants of patients with knee OA resulted in a decrease of pro-inflammatory M1-like macrophages and enhancement of anti-inflammatory M2-like macrophages [69]. Rabbits with OA have a decrease in cartilage degeneration, fewer infiltration of CD68+ cells, and less severe inflammation when provided with an intra-articular injection of mevastatin, which arrests monocyte differentiation [70]. These studies indicate that monocyte and macrophage-targeted strategies could be beneficial in OA treatment. However, timing, dosage, and delivery (locally versus systemically) are important factors to consider when applying macrophage depletion strategies, as the normal, non-chronic macrophage response is essential in tissue healing [71,72]. Moreover, monocyte depletion does not always yield a positive result for joint health, as when macrophages and monocytes are depleted systemically prior to knee joint medial collateral ligament rupture in a rat model, impaired healing and ligament strength are observed [71], and hence it should be taken into consideration. Similarly, when macrophages are depleted systemically in an obese mouse OA model, inflammation is reported to increase with infiltration of neutrophils and T cells in the joint synovium, and these mice developed OA following destabilization of the medial meniscus (DMM) surgery, demonstrating the importance of macrophages in joint homeostasis [72].

## 3. Macrophages and the Metabolic OA Phenotype

As mentioned, obesity and obesity-associated MetS are known to be key risk factors for the development and progression of OA [73,74]. Initial research into the pathophysiology of obesity-related OA highlighted a mechanical element, such as increased loading on weight-bearing joints in driving structural damage [75,76]. However, obesity has also been shown to increase the risk of OA in non-weight-bearing joints, such as the hands [77,78,79]. These various studies set forth strong evidence for a systemic influence in obesity-related OA, as mechanical stress does not explain the prevalence of OA in non-weight-bearing joints. This prompted research into adipose tissue and metabolic factors in the development of OA, and the identification of a new OA subtype, the MetS-OA phenotype [25].

The metabolic dysregulation typical of MetS is considered an important driver of low-grade systemic inflammation and pro-inflammatory macrophage polarization in adipose tissue [34]. This polarization is one of the factors contributing to an increase in the production of cytokines, favoring a constant state of low-grade systemic inflammation. This inflammatory environment associated with MetS is believed to be caused by an increase in systemic glucose and lipid levels, obesity, and hypertension, via several mechanisms [80]. First, systemic hyperglycemia can directly affect the glucose levels in cartilage, which may affect chondrocyte metabolism. High glucose levels were found to induce ROS production in chondrocytes from patients with OA due to impaired GLUT-1 downregulation, hampering optimal chondrocyte function [81]. In addition, hyperglycemia is known to induce the production of advanced glycation end products (AGEs), which upon binding to AGEs receptors, activate NF-kB and p38/MAPK signaling pathways, upregulating MMP and TNF expression [82]. AGEs are also found to increase the stiffness of the collagen network in cartilage, which alters the mechanical properties of cartilage and thereby potentially contributes to OA pathology [83]. Next, dyslipidemia, marked by increased triglycerides and free fatty acids, is a key contributor to oxidative stress. Free fatty acids induce increased production of ROS, such as superoxide radicals, hydrogen peroxide, and the reactive nitrogen species in human chondrocytes [84]. Oxidative stress contributes to OA pathology by increasing inflammatory responses and inhibiting glycosaminoglycan and collagen synthesis [85]. In addition, obesity-induced insulin resistance can affect cartilage matrix synthesis [86]. Chondrocytes express the insulin receptor, which is downregulated in chondrocytes from patients with OA and shows reduced Akt-mediated activation capacity, negatively affecting glucose transport and, thereby, the cell energy metabolism, which is essential for basic chondrocyte functions, like glycosaminoglycan production [86].

Lastly, hypertension leads to vessel contraction, compromising blood supply to the synovium, which could induce hypoxia, which is known to trigger macrophage polarization [87]. In human synovial tissue, hypoxia was found to induce M1-like macrophage gene expression via PADI4 expression, which encodes for peptidyl arginine deiminase 4, and it is known to play a role in macrophage development leading to inflammation [88]. Additionally, subchondral bone hypertension can increase intraosseous pressure and induce bone remodeling and vascularization of the cartilage. Recent findings show that the mechanism via which hypertension contributes to OA development might be sex-dependent, as in a surgically induced OA rat model with spontaneous hypertension, female rats showed more cartilage loss and synovitis in response to hypertension, while males showed a greater effect on subchondral bone remodeling compared to normotensive controls [89]. Altogether, these MetS-related mechanisms contribute to low-grade inflammation and joint degradation, initiating and contributing to the progression of OA [25,80].

When focusing on adipose tissue itself, obesity is known to cause the remodeling of adipose tissue, leading to marked changes in the function of this tissue [90]. Adipose tissue is an important and complex endocrine organ that, besides adipocytes, contains many other cells like fibroblasts, macrophages, lymphocytes, and neutrophils [91]. In patients with MetS, adipose tissue shows an increase in the release of pro-inflammatory cytokines, which is known to be primarily caused by macrophages [92]. In healthy individuals, normal adipose tissue contains approximately 5% macrophages, called resident adipose tissue macrophages, resembling M2-like macrophages by functioning to resolve inflammation, regulate lipid metabolism, and maintain homeostasis [91,93]. However, during obesity, the percentage of macrophages increases, up to 50% of total cells, with obese mice showing a phenotype switch from anti-inflammatory M2-like to predominantly pro-inflammatory M1-like macrophages [94,95]. Activated adipose tissue is seen to increase the synthesis of pro-inflammatory cytokines, such as IL-6, IL-1, and TNF, while regulatory cytokines, like IL-4 and IL-10, are decreased [96].

**Figure 1:**
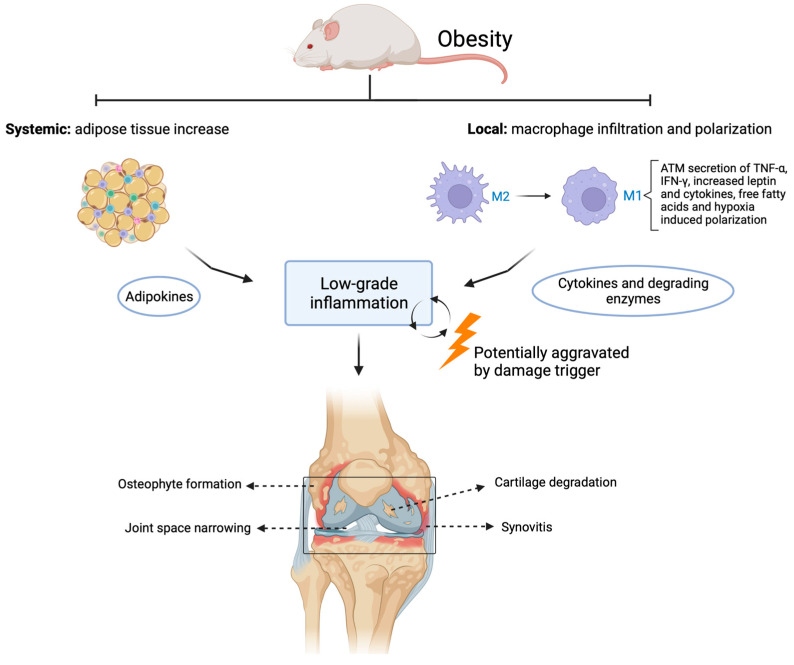
Contributing factors of obesity in metabolic OA. Systemically, adipocyte tissue increases, followed by a heightened adipokine release. In the joint, monocytes infiltrate, and a polarization switch occurs in macrophage phenotype from M2- to M1-like macrophages leading to increased release of pro-inflammatory cytokines and degrading enzymes. Both systemic and local factors contribute to a chronic state of low-grade inflammation that can lead to OA development, which is potentially further aggravated once damage occurs in the joint. ATM: adipose tissue macrophages. Created with BioRender.com, accessed on 9 March 2023.

Adipocytes also produce adipokines, a type of cytokine known to promote synovial inflammation, attract cartilage-degrading enzymes, and enhance bone matrix remodeling. Adipokines have been shown to accumulate in joints such as the knee, hip, elbow, and ankle with aging and obesity [97,98]. Adipokines such as leptin, adiponectin, and resistin are known to be associated with joint degradation and are thought to drive chronic inflammatory processes [99,100]. Leptin is associated with increases of MMP-9, MMP-13, and pro-inflammatory 1L-1β in chondrocytes from OA patients, suggesting a catabolic role in cartilage metabolism [99,101]. Both adiponectin and leptin can upregulate IL-8 production in chondrocytes [102]. IL-8 is known to increase MMP-1 and MMP-13 protein levels and was found to induce chondrocyte hypertrophy and cartilage matrix calcification, which can contribute to cartilage damage [103]. In addition, leptin was found to upregulate ADAMTS gene expression via MAPK and NF-κB signaling, which can lead to increased matrix degradation and joint inflammation [104]. In human synoviocytes, adiponectin was found to enhance IL-6 production by activating the AdipoR1 receptor via AMPKα1/p38/IKKαβ, resulting in enhanced binding of p65 and p50 to the NF-κB site and transactivation of IL-6 gene expression [105]. In synovial fluid from OA patients, visfatin is correlated with cartilage matrix degradation markers CTX-II (as a marker of collagen II) and AGG1 (as a marker of aggrecan) [106]. A specific visfatin receptor has not been identified; however, it can bind to the insulin receptor and is associated with several signaling pathways mediated by IL-6 and increased MMP and ADAMTS production [107]. In addition, adipokines may trigger macrophage switch and proliferation in the synovium, enhancing pro-inflammatory cascades and stimulation of MMP activity, contributing to cartilage degeneration [99,108]. There is much evidence of the role of adipose tissue-derived inflammatory mediators in dysregulating joint metabolism (Figure 1).

A recent study explored the role of adipose tissue in OA using a transgenic mouse model of lipodystrophy, a disorder in which the body is unable to produce adipose tissue and hence completely lacks adipokine signaling [98]. Despite the lack of adipose tissue, these mice have a body mass similar to wild-type mice. Lipodystrophy mice fed a high-fat diet were found to be protected from both spontaneous and post-traumatic (DMM) cartilage damage. However, once a small fat pad was implanted subcutaneously, this protection was lost. Hence, this showed a direct effect of adipose tissue signaling on cartilage damage, independent of body mass, increases in mechanical loading due to increased body mass, and nutrition. Interestingly, the lipodystrophy mice also showed less pain-associated behavior in response to DMM surgery compared to wild-type mice, both on a chow and high-fat diet [98]. This showed that adipose tissue might have a direct effect on OA development and joint pain-associated behavior via adipose tissue signaling, creating a state of low-grade systemic inflammation and contributing to joint degeneration and pain induction.

In addition to subcutaneous and visceral adipose tissue contributing to OA development, adipose tissue in the joint itself is also investigated as a potential contributor to OA. When looking at characteristics of adipose tissue in the knee joint of individuals with OA, it was found that adipocytes from the infrapatellar (Hoffa’s) fat pad (IPFP) of OA patients with obesity were significantly larger than adipocytes in lean OA patients along with the synovium displaying more fibrosis [109]. The IPFP is proposed to be a major source of adipokines in the knee, and macrophages are the predominant immune cell in the IPFP [97,110]. The proximity of the IPFP to other joint tissues, such as the bone, cartilage, and synovium, enables it to alter its function biochemically by adipokine signaling [111]. It was shown that high-fat feeding in mice primed the IPFP to a more inflammatory state, and in combination with OA-inducing surgery, the IPFP showed increased crown-like structures, marking macrophage-related inflammation and fibrosis [112]. Similar observations were made in rat knee joints when OA was surgically induced while rats were fed a high-fat diet, where knee joints presented greater macrophage infiltration, synovitis, and osteophytes [113]. When Wistar rats were fed a high-carbohydrate, high-fat diet, this resulted in increased synovitis and macrophage infiltration in the knee joint, with predominantly pro-inflammatory M1-like macrophages [114]. The increased pro-inflammatory macrophages as a result of obesity were considered to be one of the main drivers of both the establishment and progression of obesity-associated OA.

Animal models of obesity have been widely used to study human MetS and related diseases and appear to parallel metabolic dysregulations and low-grade inflammation as observed in human MetS [115]. Key mechanisms in high-fat diet-fed animals contributing to metabolic dysregulation are increased serum glucose, insulin, and triglycerides, increased body mass and/or adipose tissue, and elevated blood pressure [115,116]. These factors were shown to negatively affect organs like the liver, adipose tissue, and muscle, which are the major metabolic organs that are affected in humans with MetS [115]. In addition, many animal models are known to develop joint damage in response to an obesity-inducing diet alone [117,118,119,120,121]. Nonetheless, it has become clear that much variation exists between diet-induced OA models; for example, when various C57BL/6J-based OA mouse models are compared to wild-type mice and genetically modified human C-reactive protein (hCRP), low-density lipoprotein receptor-deficient (LDLr−/−.Leiden) and ApoE*3Leiden.CETP mice.5 [117]. In all these models, a high-fat, or other obesity-inducing diets, consistently induces metabolic dysregulation. However, the degree of cartilage degradation, synovitis, and osteophytosis is variable between the mouse models and variable within the sexes in the same mouse strain.

Moreover, after at least 24 weeks of high-fat diet exposure, mouse models of genetically modified hCRP (male) and ApoE*3Leiden.CETP mice.5 developed cartilage degradation, while male LDLr−/−.Leiden and wild-type mice did not, even after 52 weeks of a high-fat diet [117]. In the two humanized mouse models that did develop cartilage degeneration, this was dependent on sex, as the hCRP strain male mice developed cartilage damage while female mice did not [117]. In the ApoE*3Leiden.CETP mice, female mice developed severe cartilage degeneration dependent on the diet cholesterol supplementation, while in male mice, cartilage damage was limited to surface fibrillation and seemed completely independent of the diet and cholesterol supplementation [117]. These studies indicate that models using obesity-inducing diets are not guaranteed to develop OA and that OA development in these models can be co-dependent on factors like sex, genetics, or additional surgical damage. Nevertheless, once OA development is triggered, obesogenic diets seem to accelerate OA progression, possibly by disrupting joint homeostasis and increasing local inflammation.

In addition to macrophages located in the joint, the circulating precursors of macrophages, monocytes, could also contribute to OA development. Monocytes have been classified into three primary subpopulations, the classical, intermediate, and non-classical subtypes [122]. During homeostasis, the non-classical subtype is predominant, whereas, during many pathological conditions, such as infection, asthma, obesity, and rheumatoid arthritis, the classical and intermediate subtypes are found to be increased [122]. In patients with OA, it was found that intermediate monocytes are enriched in the synovial fluid compared to the circulation and that higher levels of intermediate monocytes correlate with worse KOOS and WOMAC function scores [123]. In mice fed a high-fat diet, increased monocyte activation was seen in addition to increased joint damage, with long-term feeding showing increased levels of systemic intermediate monocytes [124]. Increased intermediate monocyte levels could influence OA development via differentiation to pro-inflammatory macrophages in the synovium and IPFP, but also via differentiation to osteoclasts in the subchondral bone [125]. As osteoclasts derived from intermediate monocytes are described to form increased numbers of osteoclasts with a high bone resorption capacity, they could thereby disrupt healthy bone remodeling and contribute to subchondral lesions, cortical plate perforations, and OA development [125,126].

## 4. Macrophage-Related Biomarkers and Their Therapeutic Applications in OA

Biomarkers allow for the detection of initial signs of pathogenic processes and are of several types, such as radiographic, physiological, histological, or molecular (for example, protein, RNA, DNA, and metabolite-based biomarkers) [127,128]. The plethora of evidence that inflammation and pro-inflammatory factors are key to the pathological onset and progression of OA has led to increased interest in the identification of biomarkers during early signs of inflammation to manage OA in its early stages. Due to the central role occupied by macrophages in joint tissue inflammation and repair, soluble macrophage-related biomarkers that can be detected in systemic circulation upon macrophage activation might be a good strategy, as they allow for the understanding of the dynamic nature of macrophage populations in joint disease regulation as well as identification of their interactions with regulatory molecules in OA pathogenesis. Macrophage-derived markers might be a key component in understanding the early biochemical changes taking place in joint tissues via analysis of blood or synovial fluid. In addition, macrophage imaging would allow for non-invasive monitoring of macrophage-related biomarkers. If successful, macrophage-based biomarkers could reflect the abundance and inflammatory activation state of macrophages and can be indicative of the structural progression of OA and inflammation-related pain, eventually contributing to finding new strategies to improve patient care.

Over the last decade, OA biomarkers were characterized according to the BIPEDS classification, including burden of disease (B), investigative (I), prognostic (P), efficacy of intervention (E), diagnostic (D), and safety (S) [129]. For a broad overview of the current position and main OA biomarkers in development, we refer to work by the Foundation of NIH OA Biomarkers Consortium and OARSI/FDA osteoarthritis biomarkers working group [130,131,132]. In this review, we discuss some of the most promising macrophage-related biomarkers, such as folate receptor (FR), a cell surface glycosylphosphatidylinositol (GPI)- anchored glycoprotein found attached to cell membranes, functioning to bind folate (folic acid) [133,134]. FRs four isoforms consist of α, β, γ, and δ, amongst which FR-α, FR-β, and FR-γ have been identified in humans [133,135]. The FR-β marker protein is expressed by cells in the placenta, spleen, myeloid cells, and neutrophils [136,137]. FR-β has a high binding affinity for folic acid, and its elevated expression has also been studied in cancerous cells, such as acute myelogenous leukemia (AML) cells and chronic myelogenous leukemia (CML) cells [133,137,138]. In particular, several studies have identified FR-β as highly overexpressed in activated, not resting, macrophages, which are commonly involved in the pathogenesis of inflammatory and autoimmune diseases [136,139]. Its selective overexpression in activated macrophages allows precise targeting of folate-linked imaging and therapeutic agents in FR-β positive macrophages [140,141], as well as quantification of the degree of inflammation from the uptake of folate-linked imaging agents [141,142].

Earlier studies on other inflammatory diseases, such as rheumatoid arthritis, showed elevated levels of FR-β in synovial macrophages of inflamed human joints [143]. Folate-receptor expression has been shown in human studies using radiopharmaceutical folate, which binds to the FR and is taken up by activated macrophages [144]. FR-β shows promising results as a biomarker for OA in human and OA animal imaging studies, where macrophage activation is identified using radiolabeled folates [145,146,147,148]. In particular, with the use of radiolabeled folates, folate receptor expression can be traced in-vivo in humans and animals using high-resolution imaging techniques like Positron Emission Tomography (PET) and single photon emission computed tomography (SPECT) [145,149]. Using folate receptor-based SPECT-CT imaging, activated macrophages were found in vivo in most (76%) knee joints in patients with symptomatic OA [146]. In addition, macrophage activation in the knee was also positively correlated with knee pain severity, OA progression, joint space narrowing, and osteophyte formation [146]. Studies employing non-radiolabeled folate-based imaging also showed the presence of active macrophages in rat models during the early stages of OA, therefore suggesting a role of the macrophage in OA onset and progression [145]. Furthermore, immunohistochemistry and immunofluorescent microscopy show FR-β expression predominantly in synovial macrophages, indicating that in vivo FR-β imaging could contribute to the detection of early synovitis [147,148]. Folate receptor expression and macrophage activation were observed in vivo in Wistar rats using a new folate conjugate with albumin-binding entity (cm09). This folate-based imaging revealed that macrophage activity increased due to a high-fat diet combined with OA-inducing surgery [148]. In addition, an increase in macrophage activity was detected in response to papain and OA running models, which correlated with disease severity in untreated OA [150], suggesting that FR is a potential candidate as a biomarker for OA disease severity.

Besides application as a marker of disease status, FR-β also shows potential as a marker for treatment response to corticosteroids, such as triamcinolone acetonide, which was shown to be effective in combating symptoms of OA in rats by altering macrophage activation [151]. For example, triamcinolone acetonide has been shown to hinder osteophyte formation and protect against cartilage degeneration in a monosodium iodoacetate guinea pig OA model, hence helping manage symptoms of pain [152]. In a mouse model of papain-induced OA, FR-β targeted SPECT-CT showed that treatment with triamcinolone acetonide reduced osteophyte formation and enhanced FR-β^+^ macrophage activation [150]. This enhanced FR-β signal seemed to have occurred due to triamcinolone acetonide inducing anti-inflammatory monocyte differentiation and hence increasing the proportion of CD163^+^ and FR-β^+^ M2 type anti-inflammatory macrophages [150]. In vitro monocytes treated with triamcinolone acetonide skewed monocytes towards an FR-β-expressing, anti-inflammatory phenotype [153]. Without corticosteroid treatment, M-CSF was found to induce high FR-β expression that remains high under pro-inflammatory conditions, thus representing the pro-inflammatory FR-β+ macrophages that are observed in rheumatoid arthritis and osteoarthritis patients [153]. This indicates the potential of FR-β as a biomarker to monitor anti-inflammatory macrophage response to treatment with corticosteroids.

Other promising biomarkers for early OA detection have been investigated in clinical studies, such as synovial fluid and serum-based biomarkers following acute injury to the anterior cruciate ligament (ACL) [154]. Injuries to the ACL are a major risk factor for the development of OA and, consequently, knee pain [155]. Various inflammatory cytokines, matrix-degrading enzymes, and other protein concentrations were studied at two time points: at the time of acute knee injury and after a short period of recovery prior to surgery [154]. Results showed that at the time of injury, an initial decline of proteoglycan and non-collagenous protein (synovial fluid Cartilage Oligomeric Matrix Protein (_sf_COMP)) is observed, followed by collagen degradation with an increase in collagen biomarkers, specifically: C-terminal crosslinked telopeptide type II collagen (CTxII), C1,2C, C-terminal crosslinked telopeptide type I collagen (CTxI), and N-terminal telopeptides of type I collagen (NTx) in synovial fluid [154]. Furthermore, when employing soluble macrophage-related biomarkers, CD163 and CD14 are commonly used and measured in serum/plasma and synovial fluid and are specific in their nature as expression is limited to the monocyte/macrophage lineage [156,157]. These markers represent the shedding of soluble CD163 and CD14 proteins from activated macrophages in response to signals, such as LPS, TNF, and IFNγ, and are found to be representative of the inflammatory state of joints in OA [157]. While CD163, a member of the scavenger receptor cysteine superfamily, is known as an M2 marker, it can also be expressed in M1 macrophages [156]. CD14, a receptor for LPS-LPS protein binding, is reported to be higher in M2 than in the M1 macrophage subtype [156]. When studying these biomarkers in joint tissue inflammation, results showed that both soluble biomarkers, CD163 (in serum and synovial fluid) and CD14 (in synovial fluid), were significantly associated with increased activated macrophages in the knee joint [157]. CD163 and CD14 were also positively correlated with OA progression and osteophyte formation [157]. These results highlighted soluble biomarkers in identifying the inflammatory state of the knee joint and in OA pathogenesis.

**Figure 2:**
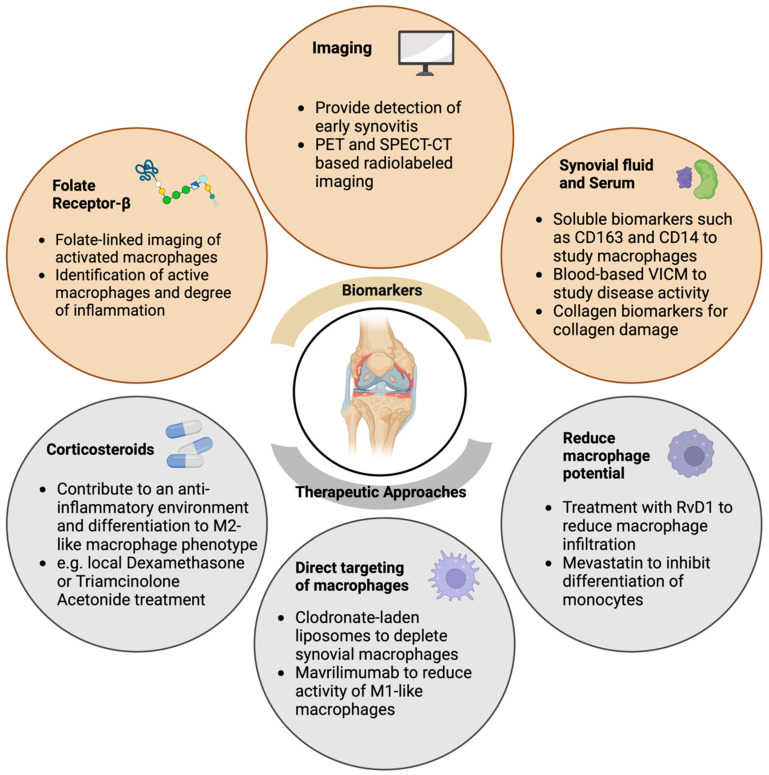
Possible candidates for macrophage-related biomarkers and therapeutic strategies in OA. Schematic representation summarizing the application of possible biomarkers and imaging candidates as well as therapeutic approaches to diagnose and treat inflammation-driven OA. PET: Positron emission tomography, SPECT: Single Photon Emission Computed Tomography, CT: Computed Tomography, IHC: Immunohistochemistry, IF: Immunofluorescence, VICM: marker of citrullinated and MMP-degraded vimentin, RvD1: resolvin D1. Created with BioRender.com, accessed on 9 March 2023.

Another blood-based biomarker for inflammation that reflects macrophage activity, and might be a good OA biomarker, is a marker of citrullinated and MMP-degraded vimentin (VICM). The biomarker VICM was shown to be released by in vitro activated macrophages and corresponded to disease activity, joint space narrowing, and joint erosions in rheumatoid arthritis [158]. Further treatment with mavrilimumab, a granulocyte-macrophage colony-stimulating factor receptor antibody (GM-CSFRα), significantly reduced VICM release and could be used to target activated macrophages [158]. A recent study explored the role of synoviocytes in OA progression by examining the molecular crosstalk between cartilage and synovium using single-cell RNA sequencing [159]. This study identified synoviocytes as a primary producer of nearly 55% of cytokines associated with OA, with a limited amount (16%) produced by chondrocytes, implying the pertinent role of synovitis in OA progression [159]. This elucidates the potential of targeting specific groups of synoviocytes for therapeutic applications by depletion or reprogramming. In addition, Sun et al. [160] observed a decrease in synovitis and cartilage destruction in obese mice upon local depletion of macrophages via clodronate-loaded liposomes in synovial joints. Furthermore, intra-articular treatment with resolvin D1 (RvD1), a pro-resolving lipid mediator capable of changing or altering the pro-inflammatory behavior of macrophages, resulted in decreased macrophage infiltration in the synovium and reduced the number of M1-like macrophages [160]. Together, this highlights the potential of targeting macrophages for therapeutic approaches to slowing OA progression. The various possible diagnostic tools and therapeutic interventions mentioned in this review are summarized in Figure 2.

## 5. Conclusions

In summary, OA is far from being defined exclusively as a wear-and-tear disease, as inflammatory processes have also been shown to be implicated in the development and progression of OA. The metabolic OA phenotype comprises a substantial group of OA patients, which have in common that they present low-grade systemic inflammation as a result of metabolic disturbances. An imbalance between macrophage M1- and M2-like phenotypes are thought to be a part of the underlying mechanism of this chronic condition. Pro-inflammatory signaling drives macrophage polarization towards the M1-like phenotype, resulting in a prolonged state of inflammation, synovitis, cartilage damage, and osteophyte formation, as seen in OA. Research on macrophage-related biomarkers in OA shows potential in terms of use in early diagnostics and monitoring of disease progression. Studies have identified biomarkers such as FR-β, blood-based VICM, and other soluble biomarkers indicative of increased macrophage activation, opening up possibilities for their use in early diagnosis, monitoring of joint tissue inflammation progress, and also in drug development trials assessing treatment response. Several pre-clinical studies have successfully applied therapeutic approaches targeting the inhibition of pro-inflammatory signals or macrophage depletion strategies. These strategies show a degree of effectiveness in managing symptoms of OA, such as osteophyte formation, synovitis, and cartilage degeneration. However, ensuring that the healthy macrophage response pertinent to tissue regulation and healing is unaltered is necessary. As knowledge of the inflammatory processes involved in OA onset and progression expands, we get closer to identifying inflammatory biomarkers that will be suitable for early diagnosis in monitoring the disease progression and/or treatment strategies. Therefore, the identification of such biomarkers will be a hallmark not only in early disease detection but will also facilitate tailoring treatment.

## Data Availability

No new data were created or analyzed in this study. Data sharing is not applicable to this article.
